# Intravitreal cotrimoxazole as adjuvant therapy for active ocular toxoplasmosis: a case series and literature review

**DOI:** 10.1186/s12348-026-00571-4

**Published:** 2026-02-20

**Authors:** Seyedeh Maryam Hosseini, Amir Azadmanesh, Ghodsieh Zamani, Mehrdad Motamed Shariati

**Affiliations:** Eye Research Center, Khatam Al-Anbia Eye Hospital, Mashhad University of Medical Sciences, Gharani Boulevard, Mashhad, Iran

**Keywords:** Ocular toxoplasmosis, Intravitreal cotrimoxazole, Vitritis, Retinochoroiditis, Adjuvant

## Abstract

**Background:**

Ocular toxoplasmosis represents the most common cause of posterior uveitis worldwide and remains a major cause of visual morbidity, particularly among young and immunocompetent individuals. The purpose of this study is to assess the efficacy and safety of combined intravitreal trimethoprim/sulfamethoxazole and dexamethasone with concurrent systemic cotrimoxazole and oral corticosteroids in patients with active Toxoplasma chorioretinitis.

**Methods:**

This retrospective interventional case series included a total of seven eyes from seven consecutive patients with active necrotizing toxoplasma retinochoroiditis and dense vitritis involving zone 1. All examinations were performed by a uveitis specialist. Each patient received a single intravitreal injection of trimethoprim/sulfamethoxazole and dexamethasone, in addition to systemic cotrimoxazole and prednisolone. Clinical evaluation included best-corrected visual acuity (BCVA), intraocular pressure (IOP), grading of ocular inflammation, and fundus examination. Patients were followed weekly for one month and subsequently on a monthly basis for three months.

**Results:**

All patients demonstrated a rapid reduction in inflammation and lesion size, accompanied by improvement in BCVA, achieving a final visual acuity of 8/10 to 10/10 at one month. No ocular or systemic complications, IOP elevation, or recurrence were observed during the three-month follow-up period.

**Conclusion:**

Adjuvant intravitreal cotrimoxazole therapy appears to be a safe and effective option for vision-threatening ocular toxoplasmosis, providing rapid disease control and excellent visual outcomes.

## Background

Ocular toxoplasmosis represents the most common cause of posterior uveitis worldwide and remains a major cause of visual morbidity, particularly among young and immunocompetent individuals [1]. It is caused by *Toxoplasma gondii*, an obligate intracellular protozoan parasite capable of establishing latent infection within retinal tissue. Reactivation of dormant cysts leads to recurrent episodes of necrotizing retinochoroiditis, characterized by intense vitritis, retinal vasculitis, and choroiditis. Although typically self-limited, untreated or severe disease may result in devastating visual sequelae, including macular scarring, optic nerve damage, retinal detachment, and secondary glaucoma [2].

The therapeutic goals in ocular toxoplasmosis include eradicating active parasitic replication and modifying the associated inflammatory response to minimize retinal destruction. Conventional therapy commonly combines antiparasitic agents—traditionally pyrimethamine, sulfadiazine, and folinic acid—with systemic corticosteroids. However, limitations of traditional regimens include hematologic toxicity, sulfonamide hypersensitivity, drug unavailability, and patient non-compliance. As a result, trimethoprim-sulfamethoxazole (cotrimoxazole) has emerged as an increasingly favored first-line systemic alternative, supported by studies demonstrating comparable efficacy, easier administration, and a favorable safety profile [3, 4].

Despite optimal systemic therapy, some patients present with treatment-refractory inflammation, progressive macular or optic nerve-threatening lesions, or contraindications to systemic medication. These challenges have led to growing interest in local therapies that achieve high intraocular drug concentrations while minimizing systemic toxicity. Intravitreal clindamycin combined with dexamethasone has been shown to effectively control active disease and is now considered a well-established therapeutic option in selected cases [5, 6].

Cotrimoxazole exerts a synergistic antimicrobial effect by inhibiting sequential steps in folate metabolism and demonstrates potent activity against *T. gondii* in systemic use [7, 8]. However, to date, intravitreal administration of cotrimoxazole remains scarcely reported, and a standardized intravitreal dosing protocol has not been established. The rationale for intravitreal delivery is to bypass the blood-retinal barrier and achieve high local antiparasitic levels, potentially accelerating inflammatory control and reducing the risk of sight-threatening complications.

This study presents a case series of seven patients with active toxoplasma chorioretinitis treated with intravitreal cotrimoxazole and dexamethasone combined with systemic cotrimoxazole and oral corticosteroids. We describe their clinical presentation, treatment response, anatomical and visual outcomes, and safety profile. Additionally, a comprehensive review of current therapeutic strategies for ocular toxoplasmosis is provided, emphasizing the evolving role of intravitreal antimicrobial and corticosteroid therapy.

## Methods

This retrospective interventional case series included seven consecutive patients diagnosed with active toxoplasma chorioretinitis at a tertiary uveitis referral center. All patients presented with active necrotizing retinitis and significant vitreous inflammation (≥ 3 + vitritis), with lesions involving zone 1 and threatening the macula or optic nerve.

A comprehensive ophthalmic evaluation was performed at baseline and each follow-up visit by a uveitis specialist. Examinations included best-corrected visual acuity (BCVA) assessment using a Snellen chart, slit-lamp biomicroscopy, indirect ophthalmoscopy, intraocular pressure (IOP) measurement with Goldmann applanation tonometry, and fundus evaluation. Vitreous inflammation was graded according to the Standardization of Uveitis Nomenclature (SUN) criteria [9, 10], and lesion characteristics were documented clinically.

At presentation, each patient received a single intravitreal injection of trimethoprim (TMP)/sulfamethoxazole (SMX) (1.28 mg / 0.08 mL) combined with dexamethasone (400 µg / 0.1 mL). All patients concurrently initiated systemic therapy consisting of oral cotrimoxazole (160 mg TMP + 800 mg SMX, twice daily) and oral prednisolone (1 mg/kg daily), with corticosteroids tapered according to clinical response. Follow-up examinations were scheduled weekly for one month and monthly thereafter for three months post-injection, all conducted by the same uveitis specialist.

Treatment response was evaluated based on reduction in inflammatory scores, resolution of retinitis borders, improvement in BCVA, and stability of IOP. Safety outcomes was assessed by monitoring for injection-related complications, including endophthalmitis, retinal detachment, cataract progression, and steroid-induced ocular hypertension. The primary outcome was improvement in intraocular inflammation, and secondary outcomes included visual recovery and treatment-related adverse events.

## Results

Seven eyes from seven patients (*n* = 7) with active Toxoplasma chorioretinitis were included. The mean age at presentation was 37.4 years (range, 19–57 years), and four patients (57.1%) were female. All eyes demonstrated dense vitritis at baseline (SUN grade 3 + to 4+) and active necrotizing retinitis involving zone 1, most commonly located parafoveally or adjacent to the optic nerve head.

Baseline BCVA ranged from hand motion (HM) to 4/10. All patients completed follow-up through the three-month visit.

A marked reduction in intraocular inflammation was observed in all patients within the first week after intravitreal therapy. By the one-month follow-up, complete resolution of active retinitis with well-defined inactive borders and substantial clearing of vitritis was documented in all cases.

Progressive visual improvement was noted in all patients. At three months, BCVA ranged from 8/10 to 10/10, representing meaningful recovery even in eyes initially affected by macula-threatening lesions.

IOP remained within normal limits in all patients throughout the follow-up period, with no clinically significant elevations at any time point. No ocular or systemic adverse events related to either intravitreal or systemic therapy were identified. Specifically, there were no cases of endophthalmitis, retinal detachment, cataract progression, or steroid-induced ocular hypertension.

Clinical characteristics and visual outcomes of patients with active toxoplasma chorioretinitis treated.

**Fig. 1:**
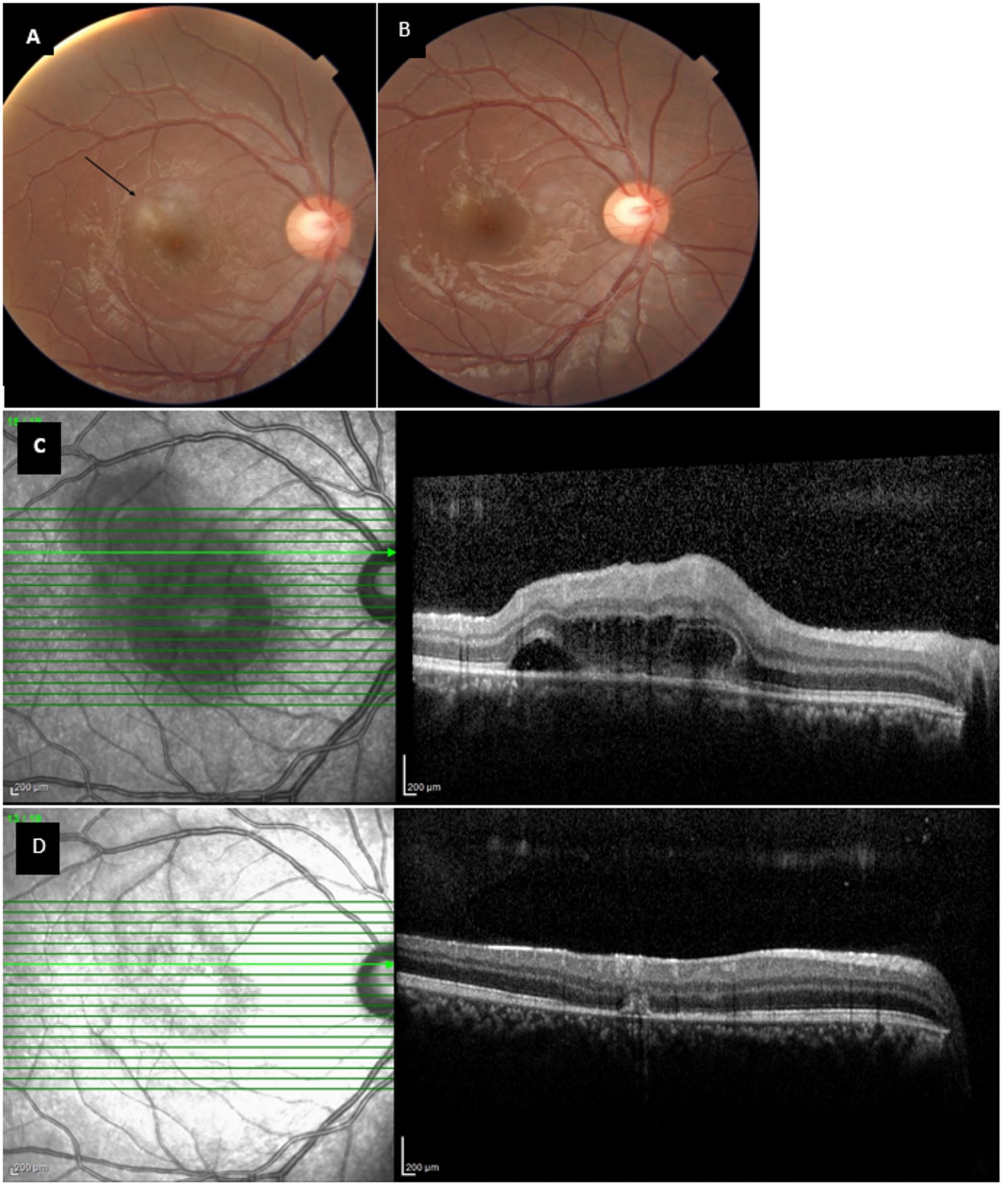
Color fundus and optical coherence tomography (OCT) images of patient 1 (**A**) Color fundus photograph at week 2 reveals marked reduction of vitritis with improved posterior segment clarity. The parafoveal necrotizing retinochoroiditis lesion exhibits decreased retinal edema, partial consolidation of borders, and reduced hyperemia compared with baseline examination. **(B)** Color fundus photograph at week 4 demonstrating complete resolution of vitritis and full inactivation of the previously active lesion, leaving a well-defined chorioretinal scar. **(C)** Raster scan OCT image of the chorioretinitis lesion at week (2) **(D)** Raster scan OCT image of the lesion at week 4 showing structural restoration and inactive chorioretinal scarring

**Fig. 2:**
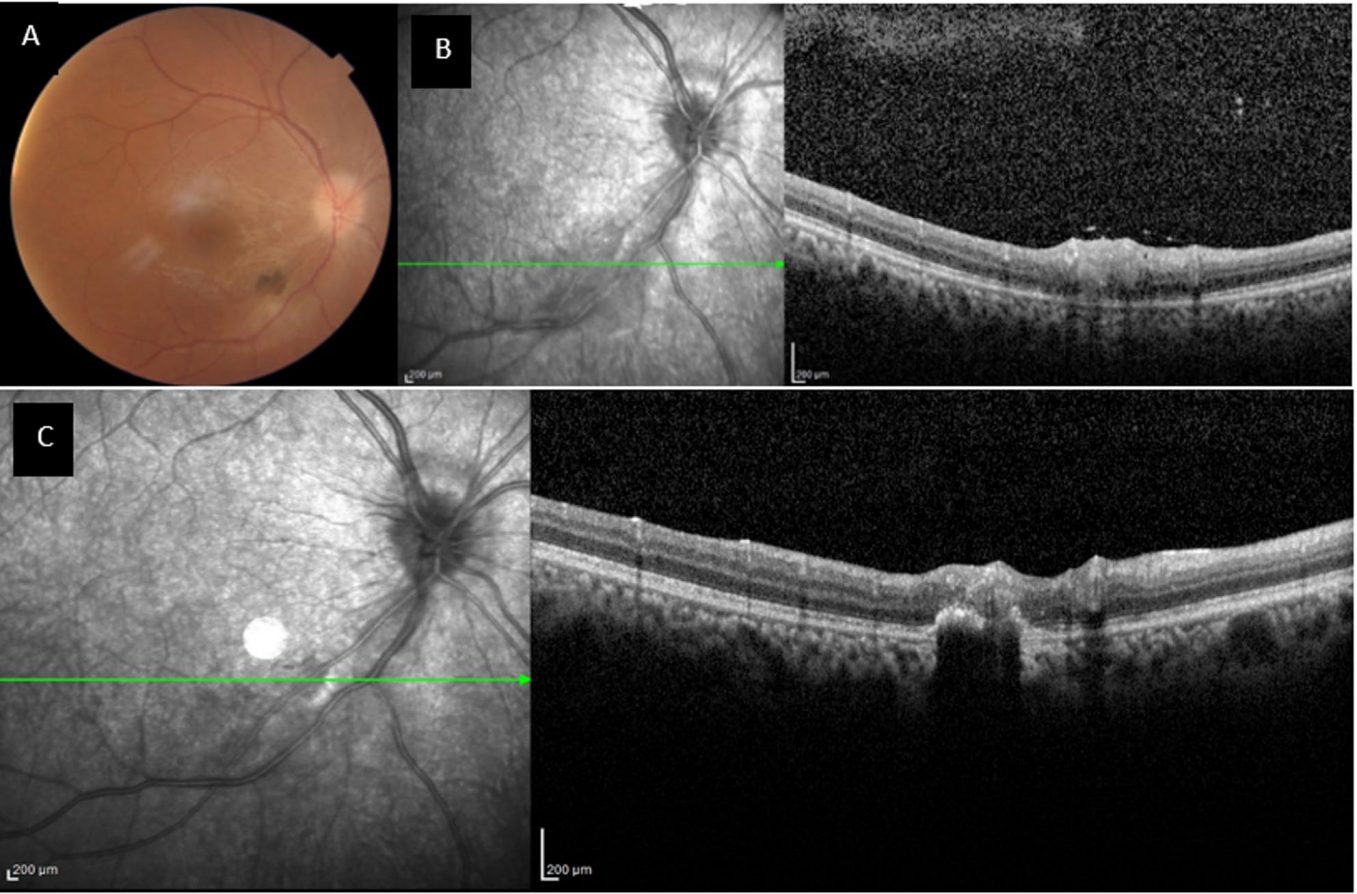
Color fundus and optical coherence tomography (OCT) images of patient 3. **(A)** Fundus photograph at week 2 shows a substantial reduction of vitreous haze and improved visualization of the posterior pole. The peripapillary retinochoroiditis lesion displays inflammatory infiltration and partial demarcation of lesion borders. **(B)** Raster scan OCT image at week 2 depicting focal retinal thickening and localized hyper-reflectivity corresponding to the active chorioretinitis lesion. **(C)** Raster scan OCT image at week 4 showing resolution of retinal edema

A summary of the clinical characteristics, visual outcomes, and IOP measurements for each patient is provided in Table 1. Representative fundus photographs and spectral-domain optical coherence tomography (OCT) raster scans from patients 1 and 3 are shown in Figs. 1 and 2, respectively. Baseline fundus photography of the eyes was not accessible due to severe vitreous haziness.

**Table 1:** Clinical characteristics and visual outcomes of patients with active toxoplasma chorioretinitis treated with intravitreal cotrimoxazole and dexamethasone combined with systemic therapy

Case	Age	Gender	Baseline BCVA	BCVA 1 week	BCVA 3 weeks	BCVA 3 month	Baseline IOP	IOP 3 month	Lesion Location	Vitritis Grade (at presentation)
1	19	M	CF 1 m	CF 3 m	4/10	10/10	13	14	Parafoveal	3+
2	52	F	HM	CF 2 m	3/10	10/10	17	19	Parafoveal	4+
3	24	F	3/10	4/10	7/10	10/10	14	16	Inferior arcade	3+
4	22	M	HM	CF 1 m	3/10	8/10	13	15	Parafoveal	4+
5	34	F	2/10	3/10	7/10	10/10	17	19	Nasal to optic disc	3+
6	56	F	CF 1 m	CF 1 m	2/10	10/10	17	19	Parafoveal	4+
7	57	F	4/10	4/10	5/10	9/10	19	19	Nasal to optic disc	3+

BCVA: Best Corrected Visual Acuity, IOP: Intraocular Pressure, CF: Counting Fingers, HM: Hand Motion

## Discussion

This case series describes the successful use of combined intravitreal trimethoprim/sulfamethoxazole (cotrimoxazole) and dexamethasone injection as an adjuvant to systemic cotrimoxazole and oral corticosteroids in patients with active *Toxoplasma* chorioretinitis. All treated eyes exhibited rapid regression of intraocular inflammation, improvement in visual acuity, and lesion resolution without complications. These findings support the potential role of intravitreal cotrimoxazole as an adjunctive therapy in vision-threatening ocular toxoplasmosis, particularly when lesions are located near the fovea or optic nerve and require a prompt, targeted therapeutic effect.

### Rationale for local–systemic combination therapy

The therapeutic goals in ocular toxoplasmosis are suppression of parasitic replication and modulation of the inflammatory response that contributes to retinal tissue damage [11, 12]. Systemic cotrimoxazole has long been used as a first-line therapy owing to its proven efficacy, favorable safety profile, and ease of use compared with classic pyrimethamine-based regimens [13, 14]. Nevertheless, despite effective systemic therapy, inflammation may persist and threaten central vision in cases with dense vitritis or macular lesions involving zone 1. The blood-retinal barrier can limit therapeutic drug levels within the vitreous—particularly early in the course of treatment—prompting consideration of intravitreal antimicrobial delivery [15].

Intravitreal administration of anti- *Toxoplasma* agents offers theoretical advantages, including immediate therapeutic concentrations at the site of infection, reduced systemic toxicity, and rapid resolution of inflammation [16]. While intravitreal clindamycin with dexamethasone has been widely adopted in selected cases, intravitreal cotrimoxazole has received limited clinical investigation [17, 18]. The favorable outcomes in our cohort support the biological plausibility of this approach: cotrimoxazole inhibits folate metabolism at two sequential steps, providing potent anti- *Toxoplasma* activity, and dexamethasone rapidly attenuates the inflammatory cascades responsible for vitreoretinal damage [19, 20].

We employed a combined regimen rather than monotherapy to leverage immediate intraocular control with intravitreal medication while maintaining systemic suppression of parasitic replication. This dual strategy may be particularly advantageous in eyes with large or centrally located lesions where early anatomical stabilization is critical.

### Visual and anatomical outcomes

All patients achieved substantial visual recovery, with final best-corrected visual acuity ranging from 8/10 to 10/10. These outcomes are notable given the macula-threatening presentation in the majority of cases and the degree of initial vitritis. Early reduction in inflammatory haze led to prompt visual improvement, consistent with a rapid onset of intravitreal drug effect. Complete lesion regression and clearing of vitritis by one month highlight the potential of this intervention to accelerate disease control. Visual recovery was not only substantial but also uniform across the cohort, indicating a high level of therapeutic consistency.

These results add to the growing evidence that timely intervention in acute *Toxoplasma* chorioretinitis—particularly when the macula or optic nerve is at risk—can prevent permanent visual sequelae. They further support the concept that achieving high intraocular drug concentrations early in the disease course may be necessary to prevent irreversible photoreceptor damage and retinal scarring [21].

### Comparison with prior studies

Previous publications have established the safety and efficacy of systemic cotrimoxazole and intravitreal clindamycin–dexamethasone for ocular toxoplasmosis, whereas reports on intravitreal cotrimoxazole remain scarce. Two clinical studies demonstrated rapid control of intraocular inflammation and favorable visual outcomes with intravitreal cotrimoxazole plus dexamethasone [22, 23]. Our findings are consistent with these early reports and further reinforce the therapeutic potential of this approach.

Compared with intravitreal clindamycin, cotrimoxazole may offer theoretical advantages, including synergistic antimicrobial activity and a broader therapeutic spectrum [24]. Systemic cotrimoxazole has trial-proven efficacy for both acute treatment and long-term prophylaxis against recurrence, whereas systemic Clindamycin does not provide sustained prophylactic benefit in toxoplasmosis [25]. In the intravitreal setting, both agents have shown excellent inflammatory control and visual recovery [26, 27]. Where clindamycin availability is limited or sulfonamide therapy is preferred, intravitreal cotrimoxazole may represent a viable alternative.

The combined local–systemic approach used in our study contrasts with regimens relying solely on intravitreal therapy. While intravitreal monotherapy has demonstrated good short-term outcomes, systemic therapy remains important to prevent recurrence. The recurrence-prevention benefit of cotrimoxazole, supported by long-term clinical trial evidence, provides additional rationale for its systemic use alongside intravitreal dosing [28, 29].

### Mechanistic considerations

The success of this regimen can be explained by several pharmacologic and immunologic mechanisms. Cotrimoxazole’s dual mechanism of action—sequential inhibition of dihydropteroate synthase and dihydrofolate reductase—provides a strong antiparasitic effect against Toxoplasma gondii tachyzoites, while its good tissue penetration allows effective intraocular levels when administered systemically. Intravitreal delivery bypasses the blood-retinal barrier, yielding immediate local drug levels, while dexamethasone alleviates host-mediated inflammation— responsible for much of the tissue damage in Toxoplasma chorioretinitis. The combined intravitreal–systemic approach thus simultaneously targets acute retinal inflammation and parasite proliferation, contributing to rapid disease resolution.

It is important to emphasize that the complete lesion regression and sustained clearing of vitritis observed at one month should not be interpreted as a direct prolonged effect of a single intravitreal antibiotic injection. The intravitreal half-life of antimicrobial agents is limited to several days, making long-term disease suppression from a single dose biologically unlikely. Instead, the primary contribution of intravitreal cotrimoxazole in this series appears to be its early, localized anti-inflammatory and antiparasitic effect, resulting in rapid reduction of vitreoretinal inflammation during the acute phase.

### Safety profile

No adverse events occurred in our cohort, including elevated intraocular pressure, cataract progression, retinal detachment, or endophthalmitis. The absence of steroid-related ocular hypertension may be attributed to short-term corticosteroid exposure and careful follow-up. This excellent safety profile aligns with prior reports and supports the tolerability of the regimen. Cotrimoxazole has well-known systemic safety advantages over pyrimethamine-based treatment, which requires hematologic monitoring [30, 31]. Intravitreal administration eliminates the risk of systemic intolerance in patients with contraindications or intolerance to oral medications; nevertheless, careful screening for sulfonamide allergy remains essential.

### Clinical implications

This treatment approach may be particularly beneficial in the following scenarios:

- Central or peripapillary lesions threaten vision and a rapid therapeutic effect is required;
- Severe vitritis obscures macular view, where early clearing aids monitoring and reduces photoreceptor injury;
- Refractory disease with inadequate response to systemic treatment;
- Resource limitation preclude use of classic agents; cotrimoxazole is widely accessible and inexpensive.

Moreover, the combination of rapid intravitreal effect and systemic prophylaxis may reduce the risk of recurrent inflammation and permanent retinal scarring — especially relevant in younger patients, who constitute the majority of ocular toxoplasmosis cases.

### Limitations

Despite encouraging findings, certain limitations merit consideration. The small sample size restricts broad generalization, and the retrospective nature of the study introduces potential selection bias. The absence of a control group precludes direct comparison with alternative treatment strategies. The follow-up period, limited to three months, primarily reflects acute treatment response; longer-term outcomes, including recurrence rates, remain to be evaluated. Although no adverse events were observed, rare complications may not be captured in small cohorts. It should be acknowledged that ocular toxoplasmosis may follow a self-limited course in certain patients, and favorable visual outcomes can occasionally be achieved with systemic therapy alone or even without treatment. In this context, it is conceivable that some eyes in our series—particularly those with relatively preserved baseline visual acuity—might have experienced improvement without intravitreal intervention. Future prospective studies with larger sample sizes, extended follow-up, and comparative designs are warranted to delineate the role of intravitreal cotrimoxazole within therapeutic algorithms.

### Perspectives and research implications

Although the present findings suggest that intravitreal cotrimoxazole combined with systemic therapy may provide rapid and effective control of active *Toxoplasma* chorioretinitis, further investigations are needed to refine its clinical role. Future studies should assess long-term outcomes, including recurrence rates and structural retinal integrity following treatment. Comparative trials assessing intravitreal cotrimoxazole against established agents such as intravitreal clindamycin would help determine relative efficacy and toxicity profiles. Additionally, pharmacokinetic studies would be valuable to clarify intraocular drug kinetics, optimal dosing intervals, and potential cumulative effects. Identification of patient-specific predictors of response may further guide personalized treatment and help select individuals most likely to benefit most from local–systemic combination treatment Table 2.

**Table 2:** Summary of key studies on the treatment of ocular toxoplasmosis

Authors (Year)	Study design	Main findings
Choudhury et al. (2015)[22]	Case series	A study was conducted to assess the effectiveness of an intravitreal injection combining trimethoprim/sulfamethoxazole and dexamethasone in treating four patients with Toxoplasma retinochoroiditis (TRC). The treatment, administered weekly or biweekly, rapidly reduced intraocular inflammation within one week, as observed both clinically and via optical coherence tomography (OCT). Remarkably, three patients achieved a 20/20 visual acuity outcome, and the remaining patient improved to 20/40 despite residual macular scarring. Furthermore, full-field electroretinogram results confirmed the treatment’s safety by showing no evidence of retinal toxicity, suggesting this combination may represent a valuable alternative treatment strategy for patients suffering from TRC.
Souza et al. (2018)[23]	Case series	This clinical study aimed to test the efficacy of an intravitreal injection combining sulfamethoxazole/trimethoprim with dexamethasone for treating active, recurrent toxoplasmic retinochoroiditis (TRC) in 13 patients whose vision was 20/63 or worse. The results demonstrated that the treatment was highly effective, leading to a visible decrease in intraocular inflammation in all eyes within two weeks. Inflammation resolved completely in the majority of patients (62%) after just a single injection, with the remaining eyes requiring a second injection. Crucially, the outcome showed the retinitis was inactive in all patients, and best-corrected visual acuity (BCVA) was either maintained or improved in every case, suggesting that this combined intravitreal drug regimen is a promising alternative therapeutic strategy for TRC patients.
Lusambo et al. (2023)[32]	Prospective cohort	A prospective study was carried out on a cohort of 54 immunocompetent Congolese patients in Kinshasa to evaluate the outcomes of using Trimethoprim/Sulfamethoxazole (TMP/SMX) as a treatment for Ocular Toxoplasmosis (OT), an inflammatory eye disease and a leading cause of infectious posterior uveitis globally, particularly where the drug is favored over the “classic” pyrimethamine/sulfadiazine regimen. The research, which tracked patient progress between February 2020 and September 2021, reported high success rates: visual acuity improved for 75.9% of the participants, and inflammation resolved in 77.5% of the cohort by the final examination. Crucially, only a small percentage (5.6%) of patients experienced a recurrence of the disease during the follow-up period, suggesting that TMP/SMX is an effective and favorable alternative treatment option for managing OT.
Felix et al. (2020)[33]	Randomized controlled trial	This randomized, double-masked clinical trial evaluated the long-term effectiveness of one year of intermittent trimethoprim-sulfamethoxazole (TMP-SMZ) therapy versus placebo in preventing recurrent toxoplasmic retinochoroiditis over a 6-year follow-up period. After 141 subjects with active recurrence initially received a 45-day course of TMP-SMZ to heal their lesions, they were randomized to receive either TMP-SMZ or placebo every other day for 311 days. The results showed a profound protective effect: the cumulative probability of recurrence after six years was a high 27.5% in the placebo group, whereas it was only 1.4% in the TMP-SMZ group (which maintained a 0% recurrence rate for the first five years). As no treatment-limiting toxicity or side effects were observed in either arm, the authors concluded that TMP-SMZ can be used safely for the long-term prophylaxis of recurrent toxoplasmic retinochoroiditis.
Alfonso et al. (2021)[34]	Meta-analysis	This systematic review and meta-analysis was conducted to assess the safety and efficacy of various existing antibiotic regimens for Ocular Toxoplasmosis (OT), given the widespread disagreement on the optimal treatment for this leading cause of posterior uveitis. Based on a synthesis of ten randomized controlled trials, the review found that while intravitreal clindamycin showed a small, statistically significant but not clinically relevant advantage in visual acuity over the classic pyrimethamine/sulfadiazine regimen, there were generally no statistically significant differences between any of the tested antibiotic schemes across other outcomes. Due to the low quality of the available evidence, the authors concluded that no single antibiotic treatment was superior to the others. Therefore, the decision for an appropriate treatment strategy in OT patients must be based on individual factors, such as the regimen’s safety profile, patient allergies to sulfa drugs, and local drug availability.
Melo et al. (2023)	Meta-analysis	The article assesses the effectiveness of direct intraocular drug administration for ocular toxoplasmosis (OT), which is the leading cause of infectious posterior uveitis worldwide. The study was motivated by the fact that conventional oral treatments for OT are frequently associated with adverse effects and often fail to prevent disease recurrence. Analyzing 52 articles, the researchers found that intravitreal injections were highly effective, resulting in improved visual acuity for 99.81% of patients treated with a combination of antiparasitic and anti-inflammatory drugs. The most commonly used drugs were the antiparasitic agent clindamycin and the anti-inflammatory agent dexamethasone. Crucially, the meta-analysis reported a very low frequency of injection-related side effects (0.49%). The authors conclude that intravitreal injections may facilitate the successful treatment of ocular toxoplasmosis and present a viable alternative, though they recommend that clinicians carefully consider a patient’s preexisting conditions.
Soheilian et al. (2005)[35]	Randomized clinical trial	This prospective, randomized single-blind clinical trial compared the efficacy of two 6-week treatment regimens for active ocular toxoplasmosis (OT): the classic triple therapy (pyrimethamine, sulfadiazine, and prednisolone) versus a combination of trimethoprim/sulfamethoxazole (co-trimoxazole) and prednisolone. The study, involving 59 patients, found that both treatments were equally effective, with all patients’ retinochoroiditis resolving after 6 weeks. Specifically, the mean reduction in lesion size was statistically similar, with a 61% reduction in the classic group and a 59% reduction in the trimethoprim/sulfamethoxazole group (*P* = 0.75). Additionally, post-treatment visual acuity improvement and the rate of disease recurrence over 24 months were also comparable between the two groups. The findings suggest that therapy with trimethoprim/sulfamethoxazole is an acceptable and effective alternative to the classic regimen for treating OT.
Yazici et al. (2009)[36]		This retrospective review investigated the use of a combination of trimethoprim/sulfamethoxazole and azithromycin along with corticosteroids as a treatment protocol for 19 patients with ocular toxoplasmosis. The results indicated that the combination therapy was highly effective: visual acuity improved by a mean of 6 ± 4 lines in 78.9% of patients, and inflammatory signs began to subside within an average of about 15 days. Over a mean follow-up period of 25 months, the recurrence rate was low at 15.8%, and the treatment was well tolerated, with only one patient (5.3%) experiencing a side effect (diarrhea). Based on these outcomes, the study concludes that the combination of trimethoprim/sulfamethoxazole and azithromycin is both an effective and safe alternative for managing ocular toxoplasmosis.

## Conclusion

In this case series, combined intravitreal cotrimoxazole and dexamethasone with concurrent systemic cotrimoxazole and oral corticosteroids resulted in rapid resolution of inflammation, significant visual improvement, and complete lesion regression in patients with active *Toxoplasma* chorioretinitis. The regimen demonstrated an excellent safety profile with no ocular or systemic complications.

These findings support the potential role of intravitreal cotrimoxazole as an effective adjunctive therapy, particularly in eyes with macula-threatening lesions or dense vitritis requiring rapid therapeutic response. While promising, these results should be interpreted in the context of the study’s small sample size and short follow-up. Larger prospective studies with extended observation periods are warranted to further validate efficacy, evaluate long-term safety, and define the optimal clinical indications and dosing strategy for intravitreal cotrimoxazole in ocular toxoplasmosis.

## Data Availability

The datasets used during the current study are available from the corresponding author on reasonable request.
